# Hepatocyte DAX1 Deletion Exacerbates Inflammatory Liver Injury by Inducing the Recruitment of CD4^+^ and CD8^+^ T Cells through NF-κB p65 Signaling Pathway in Mice

**DOI:** 10.3390/ijms232214009

**Published:** 2022-11-13

**Authors:** Hyo-Jeong Yun, Young-Joo Suh, Yu-Bin Kim, Eun-Jung Kang, Jung Hyeon Choi, Young-Keun Choi, In-Bok Lee, Dong-Hee Choi, Yun Jeong Seo, Jung-Ran Noh, Hueng-Sik Choi, Yong-Hoon Kim, Chul-Ho Lee

**Affiliations:** 1 Laboratory Animal Resource Center, Korea Research Institute of Bioscience and Biotechnology (KRIBB), Daejeon 34141, Korea; 2 Department of Functional Genomics, KRIBB School of Bioscience, University of Science and Technology (UST), Daejeon 34113, Korea; 3 School of Biological Sciences and Technology, Chonnam National University, Gwangju 61186, Korea

**Keywords:** concanavalin A, DAX1, inflammatory liver injury, NF-κB, T cell

## Abstract

Fulminant hepatitis is characterized by rapid and massive immune-mediated liver injury. Dosage-sensitive sex reversal-adrenal hypoplasia congenita critical region on the X chromosome, gene 1 (DAX1; *NR0B1*) represses the transcription of various genes. Here, we determine whether DAX1 serves as a regulator of inflammatory liver injury induced by concanavalin A (ConA). C57BL/6J (WT), myeloid cell-specific *Dax1* knockout (MKO), and hepatocyte-specific *Dax1* knockout (LKO) mice received single intravenous administration of ConA. Histopathological changes in liver and plasma alanine aminotransferase and aspartate aminotransferase levels in *Dax1* MKO mice were comparable with those in WT mice following ConA administration. Unlike *Dax1* MKO mice, *Dax1* LKO mice were greatly susceptible to ConA-induced liver injury, which was accompanied by enhanced infiltration of immune cells, particularly CD4^+^ and CD8^+^ T cells, in the liver. Factors related to T-cell recruitment, including chemokines and adhesion molecules, significantly increased following enhanced and prolonged phosphorylation of NF-κB p65 in the liver of ConA-administered *Dax1* LKO mice. This is the first study to demonstrate that hepatocyte-specific DAX1 deficiency exacerbates inflammatory liver injury via NF-κB p65 activation, thereby causing T-cell infiltration by modulating inflammatory chemokines and adhesion molecules. Our results suggest DAX1 as a therapeutic target for fulminant hepatitis treatment.

## 1. Introduction

Fulminant hepatitis (FH) is an unexpected onset of liver damage in a person with a healthy liver and the development of hepatocellular dysfunction associated with hepatic encephalopathy, coagulopathy, and jaundice [1]. If the liver is affected by multiple etiologies such as viral infection, drugs, toxins, chemicals, cardiovascular disease, or metabolic disorders [2], inflammatory responses elicit hepatocyte death signaling, which induces immune-mediated liver injury. This leads to the overproduction of cytokines from excessively activated immune cells [3] and disrupts the normal homeostasis system of the liver, consequently resulting in organ failure. Thus, appropriate activation of immune cells in the liver tissue has been associated with the maintenance of hepatic homeostasis and liver regeneration [4].

Concanavalin A (ConA), a plant lectin extracted from *Canavalia ensiformis* [5], is a mitogen that can provoke both innate and adaptive immune responses to induce hepatocyte death within a short period of time [6]. ConA-induced FH mouse models have been widely used to mimic pathological changes in autoimmune hepatitis in humans and to study the pathogenic mechanisms [7]. In these models, highly activated and recruited T lymphocytes play critical roles in the worsening of hepatic failure. For example, the treatment of BALB/c mice with T-lymphocyte-specific immunosuppressive drugs inhibited the development of hepatic damage. In addition, SCID mice with congenital immunodeficient T and B lymphocytes and athymic nude mice with immature T-lymphocyte populations showed resistance against hepatotoxic damage and protective effects following ConA treatment [8].

The dosage-sensitive sex reversal-adrenal hypoplasia congenita critical region on the X chromosome, gene 1 (DAX1; *NR0B1*), is classified as an orphan nuclear receptor without specific ligands that functions as a negative transcriptional regulator of various target genes [9]. DAX1 is responsible for regulating genes involved in the formation and development of several hormone-producing tissues in the body, such as gonads, adrenal glands, hypothalamus, and pituitary glands [10]. In our previous study, hepatocyte-specific deficiency of small heterodimer partner (SHP; *NR0B2*), which belongs to the same NR0B family as DAX1, was much more susceptible to the ConA model of hepatitis by modulating neutrophil migration in a mainly C-X-C motif ligand 2 (CXCL2)-dependent manner [11,12]. However, the effects and underlying regulation of DAX1 in inflammatory liver diseases remain largely unexplored.

Therefore, this study investigates the role of DAX1 in ConA-induced liver inflammation using cell type-specific *Dax1* knockout mice. We found that the absence of DAX1 in hepatocytes greatly contributes to the aggravation of liver damage following ConA treatment, accompanied by increased CD4^+^ and CD8^+^ T cells through the modulation of T lymphocyte-attracting chemokines and adhesion molecules, which is dependent on the NF-kB p65 signaling pathway.

## 2. Results

### 2.1. Loss of DAX1 in Myeloid Cells Does Not Affect ConA-Induced Liver Injury

**Figure 1:**
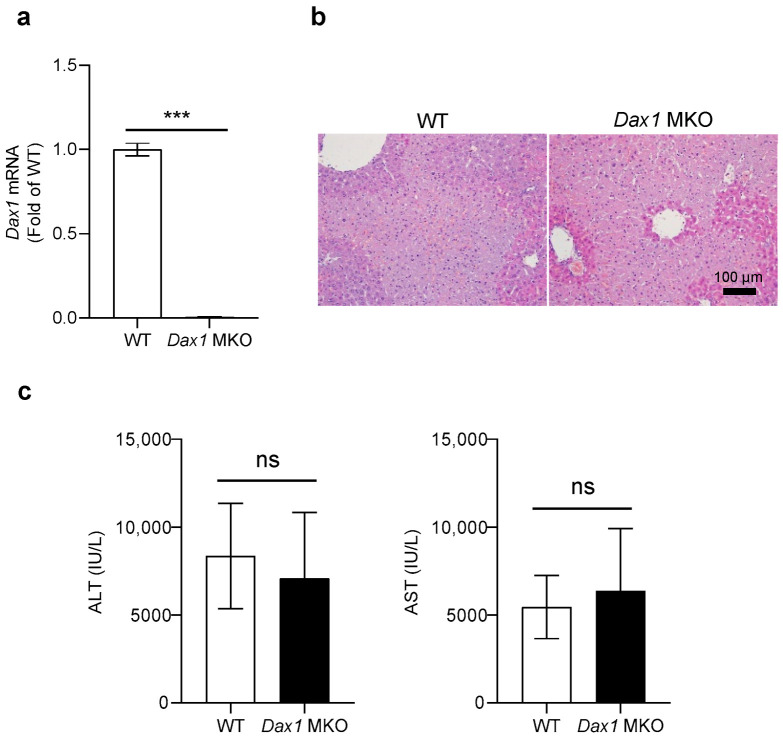
Myeloid cell-specific DAX1 deletion shows a similar effect to ConA-induced liver injury in mice. (**a**) Bone marrow-derived neutrophils from WT and *Dax1* MKO mice were isolated, and mRNA extracts from cells were conducted using quantitative real-time PCR (RT-qPCR) to measure the expression levels of *Dax1* (RT-qPCR was performed in seven technical replicates). (**b**,**c**) C57BL/6J (WT) and myeloid cell-specific *Dax1* knockout (MKO) mice were treated with single administration of concanavalin A (ConA) (15 mg/kg, i.v.) for 9 h (WT; *n* = 7, MKO; *n* = 6). (**b**) Hematoxylin and eosin (H&E) staining was performed in the liver tissues, and representative images are shown. (**c**) Plasma levels of alanine aminotransferase (ALT) and aspartate aminotransferase (AST). Data are expressed as the mean ± SEM. Two-tailed Student’s *t*-test was used to compare the MKO and WT groups; *** *p* < 0.001 and ns = not significant.

The immune response induced by ConA injection first occurs through the activation of resident innate immune cells in the liver. Among these, neutrophils, which plays an important role in mediating the recruitment of T lymphocyte, are the primary effector cells of the ConA-induced acute hepatitis model. Thus, myeloid cells, such as neutrophils, contribute to the development of ConA-induced liver injury [13,14]. For this reason, to identify how myeloid cell-specific deletion of DAX1 affects the process of ConA-induced liver injury, we produced myeloid cell-specific *Dax1* knockout (MKO) mice by crossing *Lyz*-cre mice with *Dax1*-floxed mice. To confirm the deletion of *Dax1* in myeloid cells, we assessed the *Dax1* mRNA levels of bone marrow-derived neutrophils isolated from C57BL/6J (WT) and *Dax1* MKO mice using quantitative real-time PCR (RT-qPCR). Bone marrow-derived neutrophil of *Dax1* MKO mice showed highly declined expression of *Dax1* compared to WT, which indicates that the gene of *Dax1* in myeloid cells was completely removed (Figure 1a). Next, WT mice and *Dax1* MKO mice were injected with a single ConA (15 mg/kg) dose for 9 h. Histopathological observation via hematoxylin and eosin (H&E) staining revealed that the necrotic area ranges of *Dax1* MKO mice were comparable to those of WT mice in the liver (Figure 1b). In addition, as indicators of liver damage, differences in plasma alanine aminotransferase (ALT) and aspartate aminotransferase (AST) levels between *Dax1* MKO mice and WT mice were nearly indistinguishable (Figure 1c). As indicated in Supplementary Figure S1, the expression levels of inflammatory cytokines in *Dax1* MKO mice were not significantly higher than those of WT mice following ConA treatment. These results suggest that the existence of DAX1 in myeloid cells is not involved in the ConA-induced liver immune response and inflammation.

### 2.2. DAX1 Deficiency in Hepatocytes Elevates the Susceptibility to ConA-Induced Liver Injury

**Figure 2:**
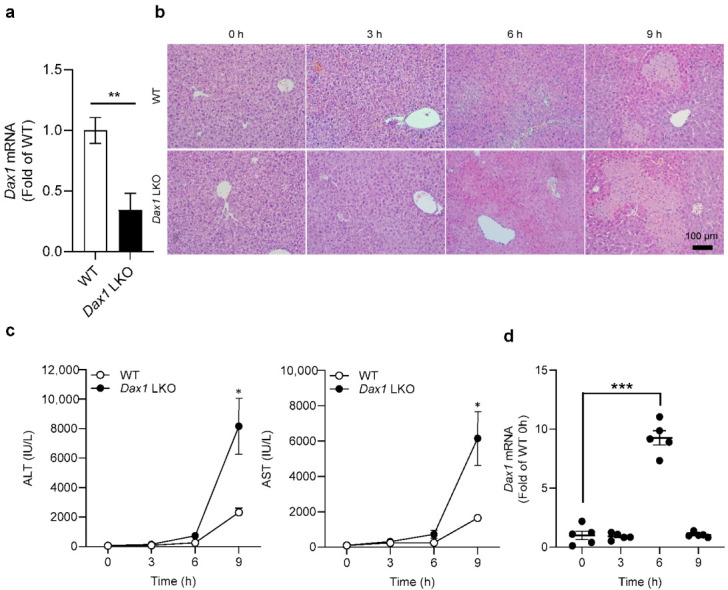
Hepatocyte-specific DAX1 deletion exacerbates the severity of ConA-induced liver injury in mice. (**a**) Primary hepatocytes from WT and *Dax1* LKO mice were isolated, and mRNA extracts from cells were conducted using RT-qPCR to measure the expression levels of *Dax1* (RT-qPCR was performed in five technical replicates). (**b**–**d**) WT and hepatocyte-specific *Dax1* knockout (LKO) mice were treated with a single administration of ConA (15 mg/kg, i.v.) for 0, 3, 6, and 9 h (WT; *n* = 5–6, LKO; *n* = 6–7). (**b**) H&E staining was performed in the liver tissues at various time points, and representative images are shown. (**c**) The plasma levels of ALT and AST were measured at the 4-time points. (**d**) The liver samples from WT mice were harvested and subjected to RTqPCR for measuring the expression levels of *Dax1* at 4-time course after ConA injection (five technical replicates for each pooled sample from WT mice). Data are expressed as the mean ± SEM. Two-tailed Student’s *t*-test was used to compare the LKO and WT groups (**a**) and the LKO groups at each time point with respective WT groups (**c**); Tukey–Kramer test after the one-way ANOVA was used to compare each time point with zero time (**d**); * *p* < 0.05, ** *p* < 0.01, and *** *p* < 0.001.

Next, to investigate the role of DAX1 in hepatocytes, which comprise over 60% of the total liver cell types in liver tissues [15], the *Alb*-cre mice and *Dax1*-floxed mice were crossed to create hepatocyte-specific *Dax1* knockout mice (LKO). To validate the generation of *Dax1* LKO mice, we identified the *Dax1* mRNA levels of primary hepatocytes derived from WT and *Dax1* LKO mice via RT-qPCR. Compared with WT, significantly decreased expression levels of *Dax1* were observed in primary hepatocytes of *Dax1* LKO mice (Figure 2a). Thereafter, WT and *Dax1* LKO mice were administered a single dose of ConA (15 mg/kg). At four different time points (0, 3, 6, and 9 h) post injection, the mice were sacrificed to assess the degree of liver damage. Interestingly, H&E staining showed that *Dax1* LKO mice exhibited a significant level of hepatic necrosis in a time-dependent manner compared with WT mice (Figure 2b). In accordance with the histopathological findings, plasma ALT and AST levels also increased over time in *Dax1* LKO mice compared with those in WT mice (Figure 2c). Additionally, the pattern of *Dax1* expression in WT mice, according to the hourly administration of ConA, was dramatically increased at 6 h compared with that at 0 h (Figure 2d). Based on these data, subsequent experiments were conducted at 6 h following ConA treatment. These results demonstrate that DAX1 deficiency in hepatocytes accelerates the susceptibility to ConA-induced liver injury in mice.

### 2.3. Hepatic Expression Levels of Inflammatory Cytokines Are Increased in Dax1 LKO Mice following ConA Treatment

**Figure 3:**
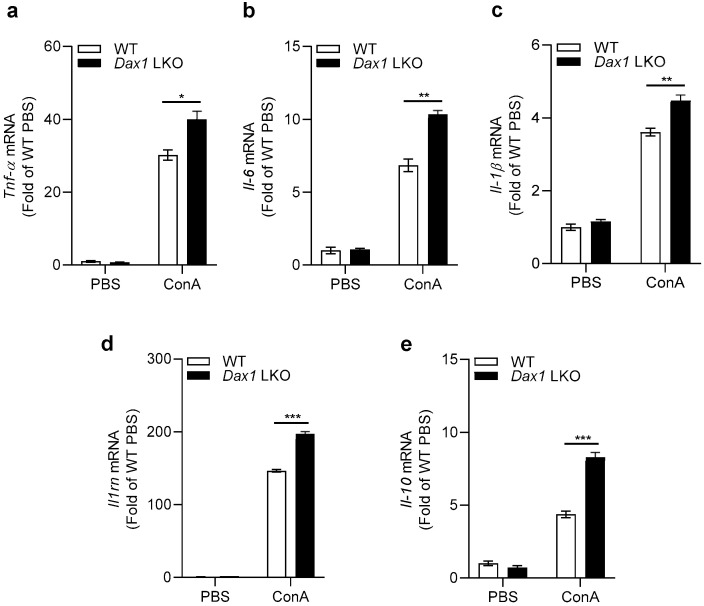
Hepatocyte-specific DAX1 deletion upregulates inflammatory cytokine expression following ConA administration. WT and *Dax1* LKO mice were treated with a single administration of ConA (15 mg/kg, i.v.) or phosphate-buffered saline (PBS). The liver samples were harvested at 6 h (PBS; *n* = 3, ConA; *n* = 6) post treatment. (**a**–**c**) Expression of pro-inflammatory cytokines (*Tnf-α*, *Il-6*, and *Il-1β*) and (**d**,**e**) anti-inflammatory cytokines (*Il1rn* and *Il-10*) determined using RT-qPCR (three technical replicates for each pooled sample from mice). Data are expressed as the mean ± SEM. Tukey–Kramer test after the one-way ANOVA was used to compare the ConA-treated LKO and ConA-treated WT groups; * *p* < 0.05, ** *p* < 0.01, and *** *p* < 0.001.

In response to ConA treatment, the inflammatory immune cells of the liver promote the release of cytokines through various inflammatory signaling pathways, which ultimately leads to acute liver failure [16]. Therefore, we examined the mRNA expression of inflammatory cytokines, including *Tnf-α*, *Il-6*, *Il-1β*, *Il1rn*, and *Il-10*, in the livers of WT and *Dax1* LKO mice 6 h post ConA exposure. Similar levels of intrahepatic expression were observed in untreated WT and *Dax1* LKO mice; however, these expression levels in *Dax1* LKO mice were significantly upregulated compared with those in WT mice after 6 h of exposure (Figure 3). These data provide evidence that DAX1 deficiency in hepatocytes strongly promotes inflammation following ConA-induced liver injury.

### 2.4. Absence of DAX1 in Hepatocytes Enhances CD4^+^ and CD8^+^ T-Cell Infiltration against ConA-Induced Liver Injury

**Figure 4:**
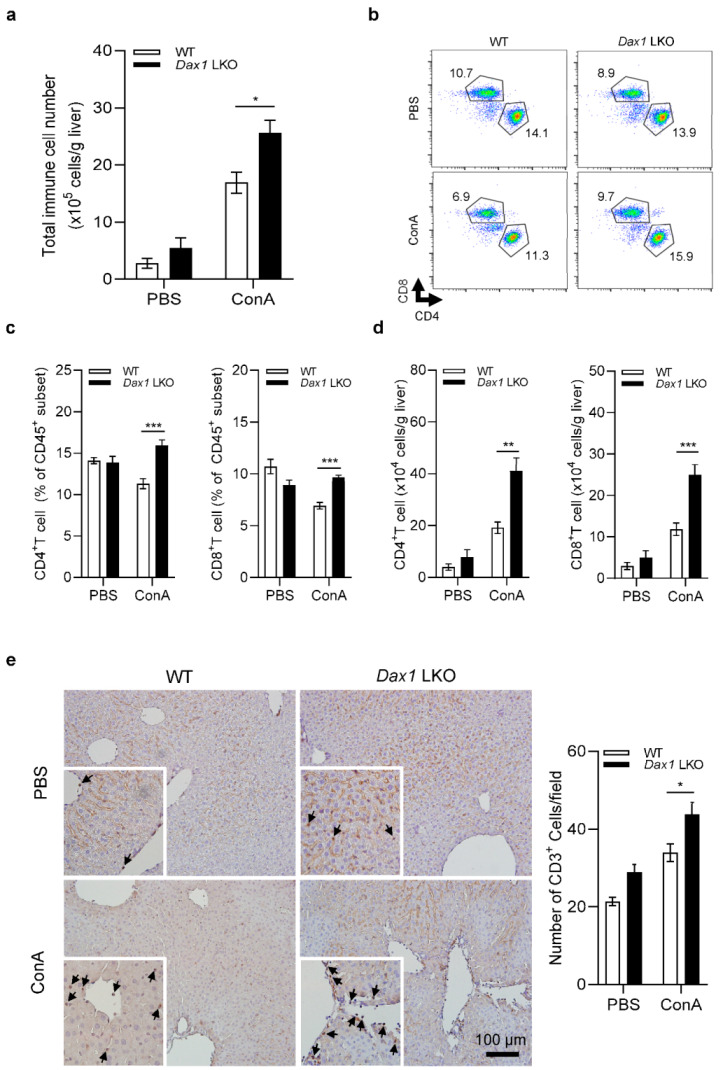
Hepatocyte-specific DAX1 deletion elevates CD4^+^ helper and CD8^+^ cytotoxic T-cell recruitment to the liver following ConA administration. WT and *Dax1* LKO mice were treated with a single administration of ConA (15 mg/kg, i.v.) or PBS. lntrahepatic immune cells were isolated at 6 h (PBS; *n* = 3, ConA; *n* = 6) post treatment and analyzed via fluorescence-activated cell sorting. Cell surface markers, including CD45, CD3e, CD4, and CD8a, were used to characterize CD4^+^ and CD8^+^ T cells. (**a**) Absolute number of whole liver immune cells (CD45^+^). (**b**,**c**) The percentages of CD4^+^T cells (CD45^+^CD3^+^CD4^+^) and CD8^+^ T cells (CD45^+^CD3^+^CD8^+^) were determined, and the representative scatterplots are shown. (**d**) Absolute number of CD4^+^ T cells (CD45^+^CD3^+^CD4^+^) and CD8^+^ T cells (CD45^+^CD3^+^CD8^+^) were determined. (**e**) Immunohistochemical liver staining for T cells (CD3, arrows) was conducted, and representative images are shown. CD3^+^ cells were counted in 4 randomly selected fields. Data are expressed as the mean ± SEM. Tukey–Kramer test after the one-way ANOVA was used to compare the ConA-treated LKO and ConA-treated WT groups; * *p* < 0.05, ** *p* < 0.01, and *** *p* < 0.001.

To determine the types of inflammatory cells involved in increasing the severity of liver damage in *Dax1* LKO mice, we conducted fluorescence-activated cell sorting (FACS) analysis with isolated immune cells from the liver 6 h following ConA injection. Although the total number of immune cells was similar in untreated WT and *Dax1* LKO mice, a markedly expanded number of immune cells were counted in *Dax1* LKO mice after ConA administration (Figure 4a). When we identified the cell population ratio in the CD45^+^ subset, the proportions of neutrophils, B cells, and iNKT cells showed no significant changes, and the infiltration of monocytes/macrophages and NK cells was significantly decreased (Supplementary Figure S2). However, the percentages of CD4^+^ and CD8^+^ T cells were notably higher in *Dax1* LKO mice than in WT mice 6 h post ConA injection (Figure 4b,c). Furthermore, the absolute numbers of CD4^+^ and CD8^+^ T cells were significantly elevated in ConA-administered *Dax1* LKO mice compared with WT mice (Figure 4d). Immunohistochemical staining with anti-CD3 antibody was performed to support the FACS analysis data. These results confirmed that a significantly higher number of CD3-positive cells were stained in the livers of *Dax1* LKO mice than in those of WT mice (Figure 4e), indicating that massive T-cell infiltration occurred in *Dax1* LKO mice. Collectively, these results indicate that CD4^+^ and CD8^+^ T cells are the key factors mediating the exacerbation of ConA-induced liver injury.

### 2.5. DAX1 Modulates the Expression of Both Chemokines and Adhesion Molecules in Hepatocytes to Promote CD4^+^ and CD8^+^ T-Cell Migration

**Figure 5:**
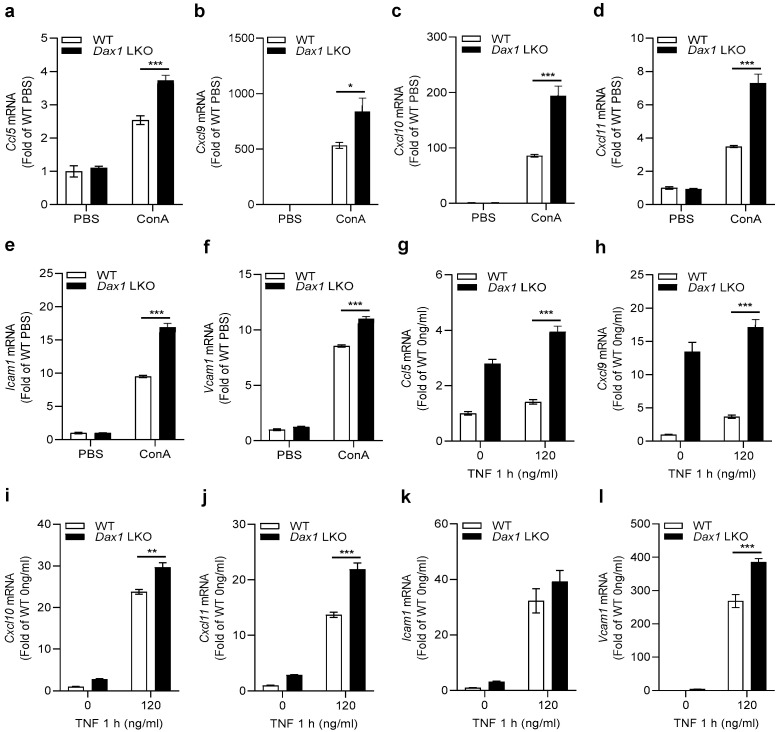
Hepatocyte-specific DAX1 deletion augments expression of chemokines and adhesion molecules related to T lymphocyte recruitment. (**a**–**f**) WT and *Dax1* LKO mice were treated with a single administration of ConA (15 mg/kg, i.v.) or PBS. The liver samples were isolated at 6 h (PBS; *n* = 3, ConA; *n* = 6) following treatment. (**a**) The expression of CC chemokine *Ccl5*, (**b**–**d**) CXC chemokines (*Cxcl9*, *Cxcl10*, and *Cxcl11*), and (**e**,**f**) adhesion molecules (*Icam1* and *Vcam1*) were examined using RT-qPCR (three technical replicates for each pooled sample from mice). (**g**–**l**) Primary hepatocytes from WT and *Dax1* LKO mice were isolated and treated with TNF-α (120 ng/mL) for 1 h. mRNA extracts from cells were conducted using RT-qPCR to measure the expression levels of *Ccl5*, *Cxcl9*, *Cxcl10*, *Cxcl11*, *Icam1*, and *Vcam1* (examinations were performed in five technical replicates). Data are expressed as the mean ± SEM. Tukey–Kramer test after the one-way ANOVA was used to compare the ConA-treated LKO and ConA-treated WT groups (**a**–**f**) and the TNF-α-treated LKO and TNF-α-treated WT groups (**g**–**l**); * *p* < 0.05, ** *p* < 0.01, and *** *p* < 0.001.

Based on the observation that DAX1 deletion in hepatocytes aggravated ConA-induced liver injury by recruiting CD4^+^ and CD8^+^ T cells, we postulated that the expression levels of genes associated with T lymphocyte attraction would increase. We measured the expression of C-C motif ligand 5 (*Ccl5*), which attracts T cells to damaged sites across the portal vessels, C-X-C motif ligand 9 (*Cxcl9*), C-X-C motif ligand 10 (*Cxcl10*), and C-X-C motif ligand 11 (*Cxcl11*), which mediate the recruitment of T cells into the parenchyma via liver sinusoids [17]. The expression of all chemokines was significantly augmented in ConA-treated *Dax1* LKO mice (Figure 5a–d). We further examined the changes in the expression of adhesion molecules that play critical roles in attracting T lymphocytes for transmigration into the liver tissue. Significantly increased expression levels of intercellular adhesion molecule 1 (*Icam1*) and vascular cell adhesion molecule 1 (*Vcam1*) were observed in ConA-treated *Dax1* LKO mice (Figure 5e,f). To determine whether the in vivo results were reproduced in an in vitro model, primary hepatocytes isolated from WT and *Dax1* LKO mice were treated with 120 ng/mL of TNF-α as a direct hepatocyte apoptosis stimulator and subjected to RT-qPCR. As shown in Figure 5g–l, *Dax1*-deleted primary hepatocytes showed significantly enhanced expression of four types of chemokines and *Vcam1*, with an increasing tendency toward *Icam1* compared to WT primary hepatocytes. Unlike the in vivo results, the TNF-α-untreated primary hepatocytes from *Dax1* LKO mice showed an increase in *Ccl5* and *Cxcl9* expression. Collectively, these results suggest that DAX1 deficiency in hepatocytes regulates the expression of chemokines and adhesion molecules related to T-cell chemotaxis, consequently boosting CD4^+^ and CD8^+^ T-cell infiltration in the liver.

### 2.6. Hepatocyte DAX1 Deletion Negatively Regulates ConA-Induced Hepatic Injury via Activation of NF-κB p65

**Figure 6:**
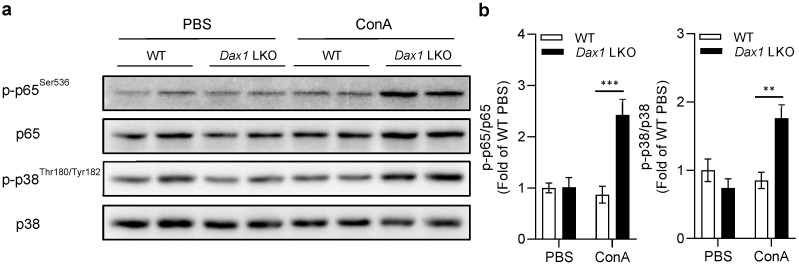
Phosphorylation of NF-κB p65 is increased in ConA-treated *Dax1* LKO mice liver. WT and *Dax1* LKO mice were treated with a single administration of ConA (15 mg/kg, i.v.) or PBS. The liver samples were isolated at 6 h (WT + PBS; *n* = 5, LKO + PBS; *n* = 7, ConA; *n* = 6) post treatment. (**a**) The protein levels of p-p65, p65, p-p38, and p38 were determined using Western blot analysis, and (**b**) the phosphorylated p65 and p38 were quantified and normalized with their respective total p65 and p38 levels. Data are expressed as the mean ± SEM. Tukey–Kramer test after the one-way ANOVA was used to compare the ConA-treated LKO and ConA-treated WT groups; ** *p* < 0.01 and *** *p* < 0.001.

We evaluated the activity of the transcription factor NF-κB p65 to determine the potential molecular mechanism of DAX1 in ConA-induced liver injury. p65 is one of the five components of the NF-κB complex, which is considered a critical mediator in inflammatory conditions and controls the expression of diverse genes, such as inflammatory cytokines (*Tnf-a*, *Il-6*, *Il-1b*, *Il1rn*, and *Il-10*), chemokines (*Ccl5*, *Cxcl9*, *Cxcl10*, and *Cxcl11*), and adhesion molecules (*Icam1* and *Vcam1*) under various stimuli [18,19]. As shown in Figure 6a,b, the ConA-treated WT mice showed nearly similar levels of p65 activation to the phosphate-buffered saline (PBS)-treated WT mice at 6 h, perhaps due to the different time points for phosphorylation. However, remarkably increased and sustained phosphorylation of p65 was confirmed in ConA-treated *Dax1* LKO mice at 6 h compared to that in ConA-treated WT mice. Next, to verify the factors that can regulate p65 phosphorylation in DAX1-deficient conditions, we focused on NF-κB inhibitor alpha (IκBα) and p38 mitogen-activated protein kinase (MAPK). The IkBα protein acts as an inhibitor of NF-κB activation in the cytoplasm by forming the NF-κB-IκBα complex, and phosphorylated IkBα dissociates from p65, leading to NF-κB activation [20]. p38 MAPK is involved in p65 upstream kinases and modulates NF-κB-driven target gene expression via the regulation of p65 phosphorylation [21,22]. Interestingly, the protein levels of phosphorylated IkBα were unaffected (Supplementary Figure S3), while the phosphorylation levels of p38 MAPK were upregulated, which closely agrees with the levels of p65 phosphorylation in *Dax1* LKO mice at 6 h post ConA treatment. Therefore, these findings suggest that DAX1 modulates the expression of chemokines and adhesion molecules by inhibiting p38-dependent excessive p65 activation in ConA-induced liver injury.

## 3. Discussion

In this study, we examined the role of DAX1 in acute inflammatory liver failure, as most investigations conducted thus far have focused on the role of DAX1 in the hypothalamic-pituitary-adrenal-gonadal axis [23]. Although previous reports have revealed the role of DAX1 in controlling hepatic gluconeogenesis and lipogenesis [24,25], further studies are required to understand its function in pathological conditions of the liver, such as hepatitis. Here, we demonstrated that loss of DAX1 in myeloid cells did not influence the development of acute inflammatory liver failure induced by ConA. In contrast, DAX1 ablation in hepatocytes stimulates the attraction of CD4^+^ and CD8^+^ T cells to the liver, accelerating the development of ConA-induced hepatitis. These findings are linked to NF-κB p65, which increased the expression of chemokines and adhesion molecules, thereby recruiting T lymphocytes.

Myeloid-lineage immune cells in the liver, such as Kupffer cells, monocytes, and neutrophils, initiate early-stage inflammation. Liver homeostasis is controlled by communication between myeloid cells and other infiltrating cells to resolve acute liver injury [26]. For this reason, we attempted to determine whether DAX1 deficiency in myeloid cells causes phenotypic changes upon ConA exposure. However, the severity of hepatic damage was not affected by DAX1 deletion in myeloid cells, implying that DAX1 plays an independent role in the direct modulation of myeloid-lineage immune cell function during inflammatory liver injury. In our previous studies, whole-body knockout mice of SHP, another member of the NR0B family with DAX1, also exhibited high susceptibility to ConA-induced hepatitis [11]. We demonstrated that SHP deficiency in liver parenchymal cells, especially hepatocytes, rather than immune cells, plays a vital role in increased liver damage following ConA treatment [12]. In the present study, hepatocyte-specific *Dax1* KO mice showed significant hepatic damage accompanied by increased plasma ALT and AST levels and dramatically elevated inflammatory cytokine expression compared with WT mice. Consistent with the SHP studies, the results of the present study indicate that DAX1 disruption in hepatocytes, rather than myeloid cells, can produce adverse effects on ConA-induced liver damage.

SHP deficiency in the whole liver resulted in highly enhanced recruitment of neutrophils and no significant changes in the population of monocytes/macrophages and T cells following ConA administration [11]. These findings have consistently been observed in hepatocyte-specific SHP-deficient mice created by the chimerization of bone marrow cells [12]. Notably, unlike our previous results from ConA-treated *Shp* KO mice [12], the proportion of infiltrated CD4^+^ and CD8^+^ T cells in *Dax1* LKO mice showed remarkable differences compared with the population of other immune cells, including neutrophils and monocytes/macrophages. These findings suggest that SHP and DAX1 play critical roles in hepatocytes, whereas the major activating immune cells in ConA-induced liver injury are distinct. We hypothesized that CD4^+^ and CD8^+^ T cells might be critical effector cells for augmented liver injury induced by ConA in hepatocyte-specific DAX1-deficient conditions.

Subsequently, to determine which factors generate enriched T cells in the liver of *Dax1* LKO mice upon ConA stimulation compared with a previous report from SHP-deficient conditions [12], we examined the alteration of gene expression related to immune cell migration. SHP ablation in hepatocytes showed increased expression of hepatic CXCL2, which is a chemoattractant for neutrophils [27], and led to massive neutrophil recruitment [12]. Meanwhile, DAX1 deficiency in hepatocytes caused elevated changes in CXCL2 expression following ConA administration (Supplementary Figure S4); however, this was insufficient to trigger excessive neutrophil infiltration (Supplementary Figure S2a). Several chemokines are known to provide signals to CD4^+^ and CD8^+^ T cells for migration to inflammation sites. The CXCL9, CXCL10, CXCL11/CXCR3 [28], and CCL5/CCR5 axes [29] mainly mediate the recruitment of CD4^+^ and CD8^+^ effector T cells during hepatic inflammation. For transmigration, the interaction between integrin expressed on lymphocytes and endothelial ligands, such as ICAM1 and VCAM1, expressed on the hepatic sinusoidal endothelium, is an essential cascade [17,30]. We observed that the expression of these chemokines and adhesion molecules was highly enhanced in *Dax1* LKO mice, in agreement with the FACS analysis. Correspondingly, the expression of four types of chemokines and two types of adhesion molecules, as well as the representative inflammatory cytokine TNF-α (Supplementary Figure S5), was elevated in TNF-α-treated *Dax1*-deficient primary hepatocytes compared with WT hepatocytes. However, the cell viability was not affected by the absence of DAX1 in hepatocytes, regardless of the concentration of TNF-α (Supplementary Figure S6). These data suggest that DAX1 in hepatocytes is strongly related to the modulation of T lymphocyte-attracting chemokine and adhesion molecule expression.

As a key mediator of immune and inflammatory responses in the liver, NF-κB controls the transcription of CCL5, CXCL9, CXCL10, CXCL11, ICAM1, and VCAM1 [31,32,33,34,35,36]. Among the subunits of NF-κB, a heterodimer of p50/p65 is the most abundant for NF-κB activation. In the canonical NF-κB pathway, phosphorylation of the inhibitory IκB protein allows for the release of p50/p65 and its nuclear translocation, facilitating the transactivation of target genes. Moreover, post-translational modifications, such as phosphorylation of p65 in trans-activation domain 1, stimulate NF-κB to bind to the sequence of the target gene promoter [37,38,39]. We found that the phosphorylation levels of IκBα in *Dax1* LKO mice were comparable to those in WT mice following ConA injection, whereas enhanced phosphorylation of the p65 subunit was identified. Furthermore, p65 is phosphorylated by numerous kinases, especially IKKα and IKKβ, to improve the transactivation potential of p65 [40]. However, our results showed that p65 activation was independently regulated by IKKα/IKKβ, with similar levels of phosphorylated IKKα/IKKβ (Supplementary Figure S7). As an upstream NF-κB regulatory kinase, p38 MAPK mediates pro-inflammatory gene expression via p65 phosphorylation [41,42]. Previous studies have observed that p38 MAPK inhibition by specific inhibitors not only attenuated the transcriptional activity of NF-κB but also reduced NF-κB-dependent gene expression [21,43]. The present study revealed that the phosphorylation pattern of p38 MAPK was similar to that of p65. Collectively, these results suggest that heightened p65 activation in the liver of *Dax1* LKO mice was irrelevant to IκBα degradation and IKKα/IKKβ activation, suggesting that p38 MAPK signaling may contribute to NF-κB activity. Further examinations in the future are needed to determine the specific mechanism by which DAX1 controls p65 activity.

In summary, to the best of our knowledge, our study is the first to identify the function of DAX1 depending on the cell type in acute liver inflammatory diseases. DAX1 deficiency causes uncontrolled NF-κB p65 activation and subsequently increases the expression of genes involved in CD4^+^ and CD8^+^ T-cell trafficking to promote excessive T-cell infiltration into the liver. This study strongly suggests DAX1 as a novel therapeutic target that could potentially elicit an anti-inflammatory effect for protection against acute inflammatory liver disease.

## 4. Materials and Methods

### 4.1. Animals Studies

*Lyz*-Cre mice, *Alb*-Cre mice, and *Dax1*-floxed mice were supplied by Jackson Laboratory (Bar Harbor, ME, USA). To obtain *Dax1* myeloid cell-specific knockout (MKO) mice, *Lyz*-Cre mice and *Dax1*-floxed mice were crossed, and *Dax1* hepatocyte-specific knockout (LKO) mice were generated by crossing *Alb*-Cre mice and *Dax1*-floxed mice. C57BL/6J (WT) control mice were obtained from the Korea Research Institute of Bioscience and Biotechnology (KRIBB; Daejeon, Korea). Eight-week-old male MKO, LKO, and WT mice were injected intravenously with a sub-lethal dose (15 mg/kg) of ConA (C2010; Sigma-Aldrich Chemical, St Louis, MO, USA). From three to seven mice per group (PBS-treated) were used at the zero-time point. At other time points, such as 3, 6, and 9 h, 5–7 mice were used per group (ConA-treated).

### 4.2. Blood Analysis

Plasma alanine aminotransferase (ALT) and aspartate aminotransferase (AST) levels were determined using an automated blood chemistry analyzer (AU480; Beckman Coulter, Brea, CA, USA).

### 4.3. Histopathology and Immunohistochemistry

Liver samples were fixed in 10% neutral buffered formalin for at least two days, embedded in paraffin, cut into 5 μm thick sections, and stained with hematoxylin and eosin (H&E). To detect T lymphocyte infiltration, liver sections were stained with anti-CD3 antibody (1:100, ab5690; Abcam, Cambridge, MA, USA) and visualized using 3,3′-diaminobenzidine (SK4100; Vector Lab, Burlingame, CA, USA).

### 4.4. Quantitative Real-Time PCR (RT-qPCR)

Total RNA was isolated from mouse livers, bone marrow-derived neutrophils, and primary hepatocytes using TRIzol reagent (Thermo Fisher Scientific, Waltham, MA, USA), and reverse transcription was performed using the UltraScript 2.0 cDNA Synthesis kit (PB30.31-10; PCR Biosystems, London, UK). The cDNA (3.5 μg/20 μL) was subjected to RT-qPCR in the StepOnePlus™ Real-Time PCR System (Applied Biosystems, Foster City, CA, USA) using the AccuPower^®^ 2 × Greenstar qPCR Master Mix (K6252; Bioneer, Daejeon, Korea) following the manufacturer’s protocol. Ct values of target genes were normalized against the Ct values of the housekeeping gene 18S rRNA, and relative gene expression levels were analyzed using the 2^−ΔΔCt^ method and compared to the control group. PCR primer sequences are listed in Supplementary Table S1.

### 4.5. Isolation of Intrahepatic Immune Cells

Liver samples collected from euthanized mice were gently and repeatedly ground using a flat-bottom 3 mL syringe plunger in ice-cold PBS and passed through a 70-μm cell strainer (BD Falcon, San Jose, CA, USA) using a sterile syringe plunger. The preparation was centrifuged at 40× *g* for 5 min at 4 °C. The supernatant was transferred to a new tube and centrifuged at 430× *g* for 5 min at 4 °C. The pellet was resuspended in 40% Percoll (17-0891-01; GE Healthcare, Buckinghamshire, UK) in PBS and centrifuged at 1265× *g* on a no-brake setting for 30 min at 4 °C. The supernatant was discarded, and erythrocyte lysis buffer (420301; BioLegend, Franklin Lakes, NJ, USA) was added to the pellet containing intrahepatic immune cells. The cells were washed once with FACS buffer before flow cytometric analysis.

### 4.6. Flow-Cytometric Analysis of Intrahepatic Immune Cells

Cells were washed with PBS, and Fc receptors were blocked with an unlabeled CD16/32 antibody (clone 93; Biolegend, Franklin Lakes, NJ, USA). The cells were washed, and extracellular marker proteins were stained for 30 min at 4 °C with fluorophore-conjugated antibodies specific to CD45 (clone 30-F11), CD3ε (clone 145-2C11), CD4 (clone RM4-5), and CD8a (53-6.7). All antibodies were procured from BD Pharmingen (San Diego, CA, USA). The cells were washed twice and analyzed using a Gallios™ flow cytometer (Beckman Coulter, Miami, FL, USA). The data were analyzed using FlowJo software (Tree-Star, San Carlos, CA, USA).

### 4.7. Bone Marrow-Derived Neutrophils

Bone marrows were collected from the pelvis, femur, tibia, and humerus of euthanized WT and *Dax1* MKO mice, mashed using pestle grind, and passed through a 70-μm cell strainer (BD Falcon, San Jose, CA, USA). Bone-marrow neutrophils were isolated via 62% Percoll (17-0891-01; GE Healthcare, Buckinghamshire, UK) density gradient centrifugation and erythrocyte lysis buffer (420301; BioLegend, Franklin Lakes, NJ, USA). Neutrophils were suspended in ice-cold Hank’s Balanced Salt Solution (14175095; Gibco, Grand Island, NY, USA).

### 4.8. Isolation of Primary Mouse Hepatocytes and In Vitro TNFα Treatment

Primary mouse hepatocytes were isolated by perfusion with collagenase type I (LS004197; Worthington Biochemical Corporation, Lakewood, NJ, USA), as previously described [44], and seeded into 24-well plates (Thermo Fisher Scientific, Waltham, MA, USA) for RTqPCR. The cells were cultured overnight in a humidified environment (5% CO_2_) at 37 °C under monolayer conditions in low-glucose Dulbecco’s modified Eagle’s medium (DMEM; WelGENE Inc., Gyeongsan-si, Gyeongsangbuk-do, Korea) containing 10% fetal bovine serum (FBS), 100 units/mL penicillin, and 100 μg/mL streptomycin. WT and *Dax1* LKO primary hepatocytes were treated with TNF-α (120 ng/mL) (PMC3016; Invitrogen, Carlsbad, CA, USA) and harvested 1 h after treatment.

### 4.9. Western Blot Analysis

Mouse liver samples were homogenized in ice-cold RIPA buffer (pH 7.4) containing 0.1 mM/L sodium vanadate, 1 mmol/L phenylmethanesulfonyl fluoride, 25 mmol/L NaF, 50 mmol/L Tris–HCl, 40 mmol/L β glycol phosphate, 120 mmol/L NaCl, 1% NP40, and 0.5% Triton X-100 added with complete protease inhibitor cocktail tablets (11836170001, Roche, Basel, Switzerland), and Xpert Phosphatase Inhibitor Cocktail Solution (p3200-010, GenDEPOT, Katy, TX, USA). Centrifugation was performed twice at 16,211× *g* for 15 min at 4 °C, and the concentration of the soluble protein fraction was determined using a Bradford assay. Extracted proteins (20 µg) were separated using 10% sodium dodecyl sulfate-polyacrylamide gel electrophoresis and subjected to Western blotting using anti-phospho p65 (Ser536) (3033S, CST, 1:1000), anti-p65 (8242S, CST, 1:1000), anti-phospho p38 MAPK (Thr180/Tyr182) (4511S, CST, 1:1000), and anti-p38 MAPK (9212S, CST, 1:1000). Proteins in the Western blots were quantified by densitometry using ImageJ 1.43 (Wayne Rasband National Institutes of Health, Bethesda, MD, USA).

### 4.10. Statistical Analyses

Numerical data are presented as the mean ± standard error (SEM). Comparisons of multiple groups were performed using the Tukey–Kramer test after one-way ANOVA when comparing three or more experimental conditions and two-tailed Student’s *t*-test when comparing two experimental conditions. Statistical significance was set at *p* < 0.05.

## Data Availability

The data that support the findings of this study are available from the corresponding author upon reasonable request.

## Supplementary Materials

The following supporting information can be downloaded at: https://www.mdpi.com/article/10.3390/ijms232214009/s1.
