# Supplementary material for: Hepatocyte DAX1 Deletion Exacerbates Inflammatory Liver Injury by Inducing the Recruitment of CD4+ and CD8+ T Cells through NF-κB p65 Signaling Pathway in Mice
Source: Int J Mol Sci. 2022 Nov 13;23(22):14009. doi: 10.3390/ijms232214009 (PMC9698938; doi:10.3390/ijms232214009)
Supplement: Supplementary file 1 [file ijms-23-14009-s001.zip › ijms-2037827-supplementary.pdf]

Supplementary Materials and Methods

1. Flow-cytometric analysis of intrahepatic immune cells

Cells were washed with PBS, and Fc receptors were blocked with an unlabeled CD16/32 antibody (clone 93; Biolegend, Franklin Lakes, NJ, USA). The cells were washed, and extracellular marker proteins were stained for 30 min at 4 °C with fluorophore-conjugated antibodies specific to CD45 (clone 30-F11), CD11b (clone M1/70), Ly6G (clone 1A8), Ly6C (clone AL-21), CD3ε (clone 145-2C11), NK1.1 (clone PK136), TCRβ (clone H57-597), B220 (clone RA3-6B2), and CD1d/αGalCer (TS-MCG-1, MBLI, Woburn, MA, USA). First five antibodies were procured from BD Pharmingen (San Diego, CA, USA) and NK1.1, TCRβ, and B220 were procured from Biolegend (Franklin Lakes, NJ, USA). The cells were washed twice and analyzed using a Gallios™ flow cytometer (Beckman Coulter, Miami, FL, USA). The data were analyzed using FlowJo software (Tree-Star, San Carlos, CA, USA).

2. Quantitative real-time PCR (RT-qPCR)

Total RNA was isolated from mouse livers and primary hepatocytes using TRIzol reagent (Thermo Fisher Scientific, Waltham, MA, USA), and reverse transcription was performed using the UltraScript 2.0 cDNA Synthesis kit (PB30.31-10; PCR Biosystems, London, UK). The cDNA (3.5 µg/ 20 µl) was subjected to RT-qPCR in the StepOnePlus™ Real-Time PCR System (Applied Biosystems, Foster City, CA, USA) using the AccuPower® 2 × Greenstar qPCR Master Mix (K6252; Bioneer, Daejeon, Korea) following the manufacturer's protocol. Ct values of target genes were normalized against the Ct values of the housekeeping gene 18S rRNA, and relative gene expression levels were analyzed using the $2^{-\Delta\Delta Ct}$ method and compared to the control group.

3. Western blot analysis

Mouse liver samples were homogenized in ice-cold RIPA buffer (pH 7.4) containing 0.1 mM/l sodium vanadate, 1 mmol/l phenylmethanesulfonyl fluoride, 25 mmol/l NaF, 50 mmol/l Tris–HCl, 40 mmol/l β glycol phosphate, 120 mmol/l NaCl, 1% NP40, and 0.5% Triton X-100 added with complete protease inhibitor cocktail tablets (11836170001, Roche, Basel, Switzerland), and Xpert Phosphatase Inhibitor Cocktail Solution (p3200-010, Gen-DEPOT, Katy, TX, USA). Centrifugation was performed twice at 16,211 × g for 15 min at 4 °C, and the concentration of the soluble protein fraction was determined using a Bradford assay. Extracted proteins (20 µg) were separated using 10% sodium dodecyl sulfate-polyacrylamide gel electrophoresis and subjected to western blotting using anti-phospho-IκBα (Ser32/36) (9246S, CST, 1:1000), anti-IκBα (4812S, CST, 1:1000), anti-IKKα/β (Ser180/181) (PA5-104696, Thermofisher, 1:1000), and anti-GAPDH (2118S, CST, 1:1000). Proteins in the western blots were quantified by densitometry using ImageJ 1.43 (Wayne Rasband National Institutes of Health, Bethesda, MD, USA).

4. Isolation of primary mouse hepatocytes and in vitro cell viability after TNF-α treatment.

Primary mouse hepatocytes were isolated by perfusion with collagenase type I (LS004197; Worthington Biochemical Corporation, Lakewood, NJ, USA), as previously described [43]. Subsequently, primary hepatocytes were seeded onto 24-well culture dishes for RTqPCR and cell viability tests. The cells were cultured overnight in

a humidified environment (5% CO_2) at 37 °C under monolayer conditions in low-glucose Dulbecco's modified Eagle's medium (DMEM; WelGENE Inc., Gyeongsan-si, Gyeongsangbuk-do, Korea) containing 10% fetal bovine serum (FBS), 100 units/ml penicillin, and 100 μg/ml streptomycin. WT and *Dax1* LKO primary hepatocytes were treated with various concentrations of TNF-α (0, 20, 40, 60, 80, 100, and 120 ng/ml) (PMC3016; Invitrogen, Carlsbad, CA, USA). After the final 9 h of culture with TNF-α, cell viability was measured using the Cell Counting Kit-8 (Dojindo Molecular Technology, Inc., Rockville, MD, USA) with 2-(2-methoxy-4-nitrophenyl)-3-(4-nitrophenyl)-5-(2,4-disulfophenyl)-2H-tetrazolium monosodium salt (WST-8).

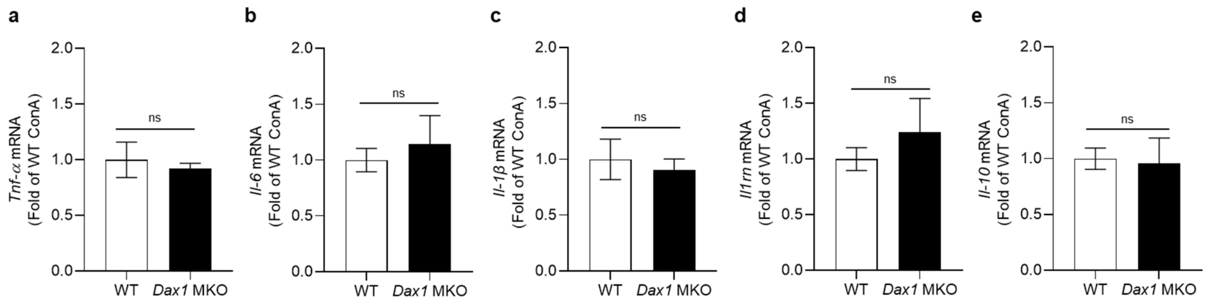

Supplementary Figure S1. Myeloid cell-specific DAX1 deletion dose not alter the expression levels of inflammatory cytokine after ConA treatment. WT and *Dax1* MKO mice were treated with a single administration of ConA (15 mg/kg, i.v.). The liver samples were harvested at 9 h (WT; n = 7, MKO; n = 6) post treatment. (**a-c**) Expression of pro-inflammatory cytokines (*Tnf-α*, *Il-6*, and *Il-1β*) and (**d,e**) anti-inflammatory cytokines (*Il1rn* and *Il-10*) determined using quantitative real-time PCR (RT-qPCR). Data are expressed as the mean ± SEM. Two-tailed Student's *t*-test was used to compare the MKO and WT groups; ns = not significant.

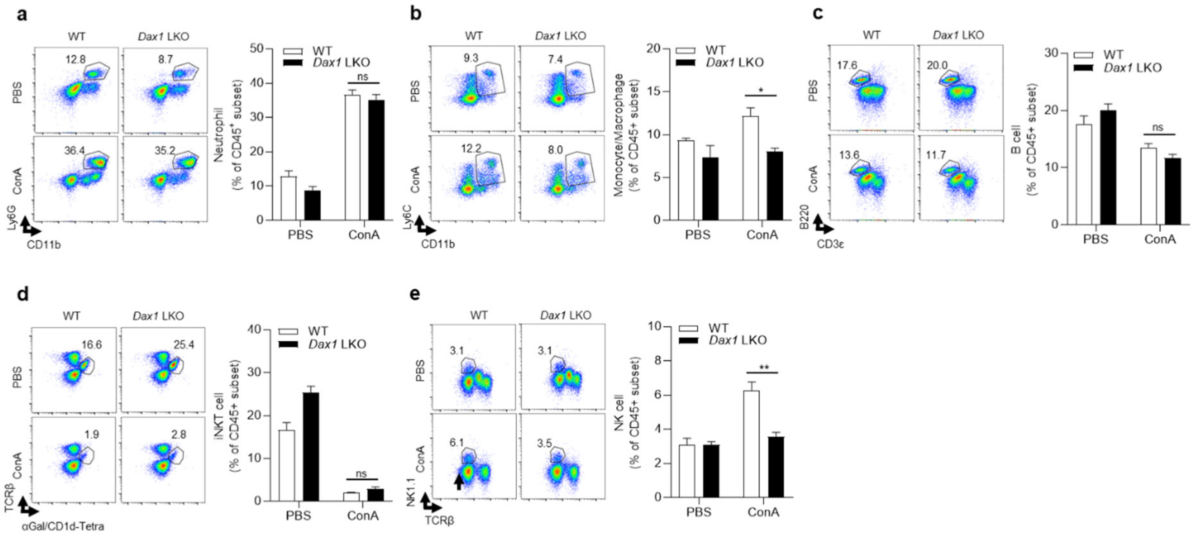

Supplementary Figure S2. DAX1 deficiency in hepatocytes induces the comparable infiltration of neutrophil, monocyte/macrophage, B cell, iNKT cell, and NK cell after ConA treatment. WT and *Dax1* LKO mice were treated with a single administration of ConA (15 mg/kg, i.v.) or PBS. lntrahepatic immune cells were isolated at 6 h (PBS; *n* = 3, ConA; *n* = 6) post treatment and analyzed via fluorescence-activated cell sorting. (**a**) The ratio of neutrophils ($CD45^+CD11b^+Ly6G^+$), (**b**) monocytes/macrophages ($CD45^+CD11b^+Ly6G^-Ly6C^+$), (**c**) B cells ($CD45^+CD3^-B220^+$), (**d**) iNKT cells ($CD45^+$αGal/CD1d-$Tetra^+TCR\beta^+$), and (**e**) NK cells ($CD45^+CD3^-TCR\beta^+NK1.1^+$) were determined, and the representative scatterplot are shown. Data are expressed as the mean ± SEM. Tukey–Kramer test after the one-way ANOVA was used to compare the ConA-treated LKO and ConA-treated WT groups; * $p < 0.05$, ** $p < 0.01$, and ns = not significant.

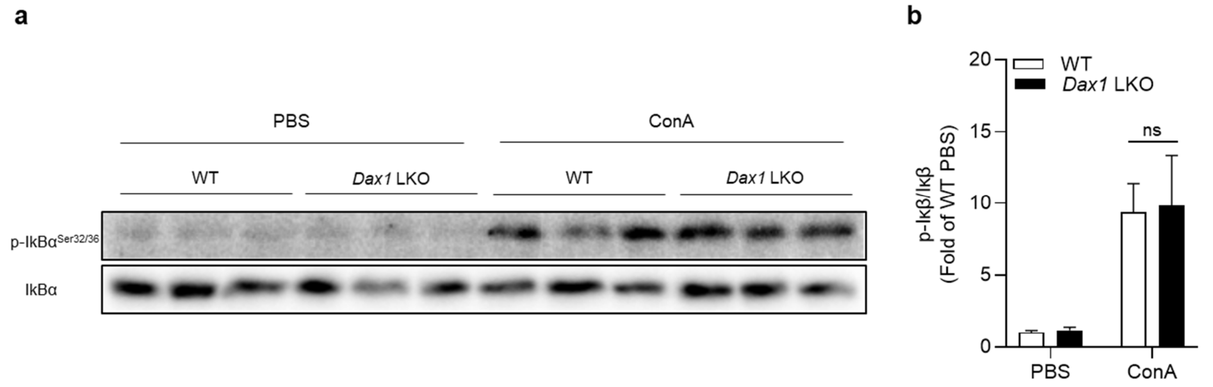

Supplementary Figure S3. DAX1 deletion in hepatocytes is unrelated to modulation of classical NF-κB pathway mediated by IkBα degradation after ConA treatment. WT and *Dax1* LKO mice were treated with a single administration of ConA (15 mg/kg, i.v.) or PBS. The liver samples were isolated at 6 h (PBS; *n* = 3, ConA; *n* = 6) post treatment. (**a**) The protein levels of p-IkBα and IkBα were determined using western blot analysis, and (**b**) the phosphorylated IkBα were quantified and normalized with total IkBα levels. Data are expressed as the mean ± SEM. Tukey–Kramer test after the one-way ANOVA was used to compare the ConA-treated LKO and ConA-treated WT groups; ns = not significant.

a

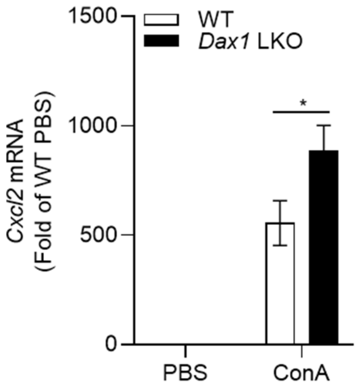

Supplementary Figure S4. Loss of DAX1 in hepatocytes affects the levels of *Cxcl2* expression after ConA treatment. WT and *Dax1* LKO mice were treated with a single administration of ConA (15 mg/kg, i.v.) or PBS. The liver samples were harvested at 6 h (PBS; $n = 3$, ConA; $n = 6$) following treatment. (**a**) The expression levels of C-X-C motif ligand 2 (*Cxcl2*) were examined using RT-qPCR (three technical replicates for each pooled sample from mice). Data are expressed as the mean ± SEM. Tukey–Kramer test after the one-way ANOVA was used to compare the ConA-treated LKO and ConA-treated WT groups; * $p < 0.05$.

a

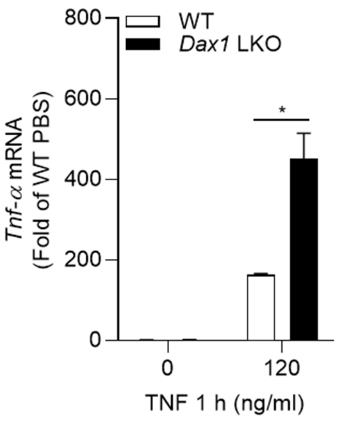

Supplementary Figure S5. Absence of *Dax1* in hepatocyte upregulates the levels of *Tnf-α* expression in response to TNF-α. Primary hepatocytes from WT and *Dax1* LKO mice were isolated and treated with TNF-α (120 ng/ml) for 1 h. (**a**) mRNA extracts from cells were conducted using RT-qPCR to measure the expression level of *Tnf-α* (examinations were performed in twice technical replicates). Data are expressed as the mean ± SEM. Tukey–Kramer test after the one-way ANOVA was used to compare the TNF-α-treated LKO and TNF-α-treated WT groups; * $p < 0.05$.

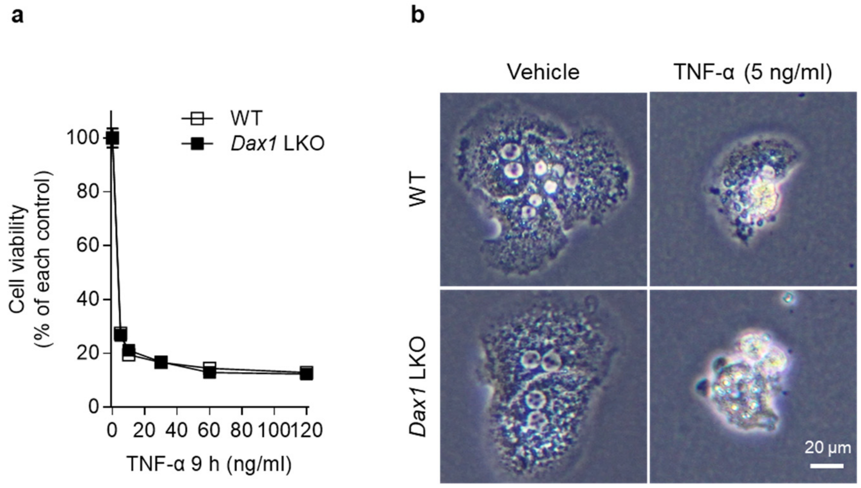

Supplementary Figure S6. Ablation of *Dax1* in hepatocyte does not aggravate the cell death in response to TNF-α. Primary hepatocytes from WT and *Dax1* LKO mice were isolated and treated with different concentrations of TNF-α (0, 20, 40, 60, 80, 100, and 120 ng/ml) for 9 h. (**a**) Cell viability was determined using a colorimetric cell viability assay kit (data were obtained from three independent experiments). (**b**) Representative images of mouse primary hepatocyte morphology are shown. Data are expressed as the mean ± SEM. Two-tailed Student's t-test was used to compare the LKO and WT groups at each concentration.

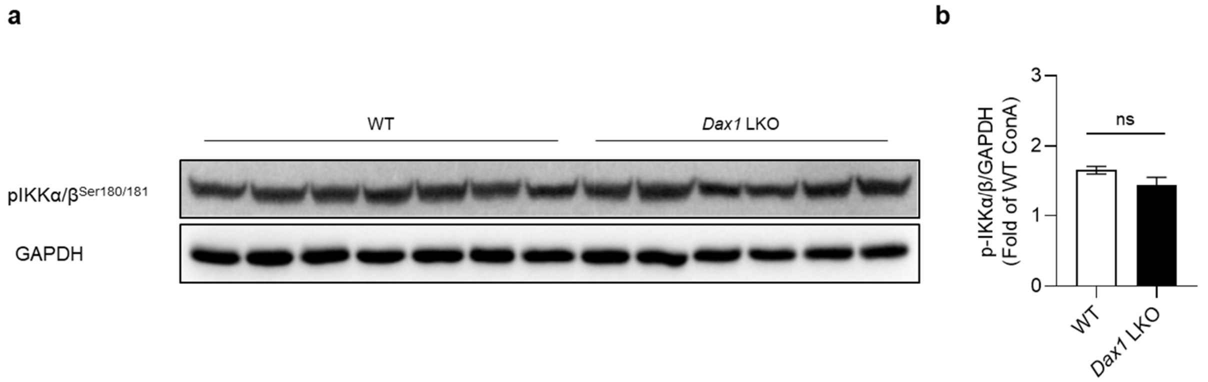

Supplementary Figure S7. DAX1 deletion in hepatocytes has no effect on IKKα and IKKβ phosphorylation after ConA treatment. WT and *Dax1* LKO mice were treated with a single administration of ConA (15 mg/kg, i.v.). The liver samples were isolated at 6 h (WT; $n = 7$, LKO; $n = 6$) following treatment. (**a**) The protein levels of p-IKKα/β and GAPDH were determined using western blot analysis, and (**b**) the phosphorylated IKKα/β were quantified and normalized with GAPDH levels. Data are expressed as the mean ± SEM. Two-tailed Student's *t*-test was used to compare the LKO and WT groups; ns = not significant.

Supplementary Table S1. Primer sequences for RTqPCR.

Gene	Primer Sequence	Gene Bank Accession number
Dax1	(F) 5'- AGATTCATCAATAGCGATGT -3'<br>(R) 5'- AGCTACGACCGCTTTCTCCA -3'	NM_007430.5
Tnf-α	(F) 5'- GGTCCCCAAAGGGATGAGAA - 3'<br>(R) 5'- TGAGGGTCTGGGCCATAGAA - 3'	NM_001278601.1
Il-6	(F) 5'- TTCCATCCAGTTGCCTTCTTG -3'<br>(R) 5'- GGGAGTGGTATCCTCTGTGAAGTC -3'	NM_031168.2
Il-1β	(F) 5'- CTACAGGCTCCGAGATGAACAAC -3'<br>(R) 5'- TCCATTGAGGTGGAGAGCTTTC -3'	NM_008361.4
Il1rn	(F) 5' - CTTGTGCCAAGTCTGGAGATGA - 3'<br>(R) 5' - CTGGACAGGCAGCTGACTCA - 3'	NM_001039701.3
Il-10	(F) 5'- GGGTTGCCAAGCCTTATCG -3'<br>(R) 5'- TCTCACCCAGGGAATTCAAATG -3'	NM_010548.2
Ccl5	(F) 5'- GACACCACTCCCTGCTGCTT -3'<br>(R) 5'- ACAAACACGACTGCAAGATTGG -3'	NM_013653.3
Cxcl2	(F) 5'- GGCTGTTGTGGCCAGTGAA -3'<br>(R) 5'- CGCCCTTGAGAGTGGCTATG -3'	NM_009140.2
Cxcl9	(F) 5'- CCCTAGTGATAAGGAATGCA -3'<br>(R) 5'- GTTCTTCAGTGTAGCAATGA -3'	NM_008599.4
Cxcl10	(F) 5'- CCCAAGTGCTGCCGTCATTT -3'<br>(R) 5'- ATTCTCACTGGCCCGTCATC -3'	NM_021274.2
Cxcl11	(F) 5'- CAAGGCTTCCTTATGTTCAA -3'<br>(R) 5'- TTTGTCGCAGCCGTTACTCG -3'	NM_019494.1
Icam1	(F) 5'- TCACCAGGAATGTGTACCTGACA -3'<br>(R) 5'- ATCACGAGGCCCACAATGAC -3'	NM_010493.3
Vcam1	(F) 5'- TGACTCCATGGCCCTCACTT -3'<br>(R) 5'- CGTCCTCACCTTCGCGTTTA -3'	NM_011693.3
18s rRNA	(F) 5'- GACACGGACAGGATTGACAGATTGATAG -3'<br>(R) 5'- GTTAGCATGCCAGAGTCTCGTTCGTT -3'	NR_003278.3
